# Lightweight Reactive Powder Concrete Containing Expanded Perlite

**DOI:** 10.3390/ma14123341

**Published:** 2021-06-17

**Authors:** Stefania Grzeszczyk, Grzegorz Janus

**Affiliations:** Department of Building Materials Engineering, Faculty of Civil Engineering and Architecture, Opole University of Technology, Katowicka Str. 48, 45-061 Opole, Poland; grzegorz.janus@doktorant.po.edu.pl

**Keywords:** lightweight RPC, expanded perlite aggregate (EP), compressive strength, density, water absorption

## Abstract

This paper presents the test results of the lightweight concrete properties obtained by adding expanded perlite (EP) to an RPC mix in quantities from 30% to 60% by volume of the concrete mix. It has been shown that in these cases it is possible to obtain concrete containing 30% by volume with density of approximately 1900 kg/m^3^ and the compressive strength > 70 MPa, with a very low water absorption value (3.3%), equal to the water absorption value of RPC without lightweight aggregate (3.3%). However, with the increased quantity of perlite (from 45% to 60%), the concrete density reduction is not observed, as the expanded perlite demonstrates very low resistance to crushing. With the increased amount of perlite, the longer periods of mixing time for all the mix components are required to obtain the homogeneous and fluid concrete mix, what causes grounding down EP. Therefore, using larger quantities of this aggregate in RPC is not recommended. The lightweight RPC shows very good freeze-thaw resistance in the presence of de-icing salt (the scaling mass is lower than 0.1 kg/m^2^). The above is explained by the compact microstructure of this concrete and the RPC mix location in open pores on the perlite aggregate surface, which consequently affects the strengthening of the aggregate-matrix contact without an interfacial transition zone (ITZ) visible. It has been demonstrated that pozzolanic activity of expanded perlite is much lower than the activity of silica fume and quartz powder, and its impact on increasing the RPC strength is minimal.

## 1. Introduction

Lightweight aggregates applied to concretes, according to the definition included in PN-EN 206+A1:2016-12 standard, have the density of <2000 kg/m^3^ in an oven-dry condition, or loose bulk density of <1200 kg/m^3^ [1]. They may be divided into natural lightweight aggregates which include volcanic tuff, scoria, or perlite, and artificial (processed) lightweight aggregates that include expanded clay, expanded perlite, expanded shale, lightweight aggregate from processed fly ash, or vermiculite [2].

Perlite is a natural volcanic rock, and approximately 97% of it is extracted in six countries (Turkey, Greece, Japan, Italy, USA, and Hungary) [3]. Perlite is an amorphous rock with a high content of silica in its composition [4]. When natural perlite is heated at 900–1200 °C, a lightweight porous material is created, so-called expanded perlite (EP) [3]. During the heating process, the base material increases its volume as much as 20 times, which leads to the production of a very lightweight porous material with outstanding thermal insulation properties and a low thermal conductivity value. Moreover, EP shows good sound-proofing properties, good fire resistance, and a high resistance to chemicals [3].

Porosity of lightweight aggregate is associated with its density, which is the main parameter that determines its suitability for use in structural concretes [5]. Expanded perlite is a very porous material. According to authors [6], the total porosity of perlite may reach up to 26 vol.%, which explains the low density of this material. Porosity of the material is also connected with its water absorption, which in case of perlite is significant and may amount to as much as 35% [7]. Due to the significant water absorption of lightweight aggregate, designing and production of lightweight concrete with those aggregate is complicated [8,9].

Expanded perlite may be used in building materials as an additive to cement (in a form of powder) or as a lightweight aggregate in thermal insulating materials. Due to its high silica and aluminate content, EP may also be the main component of geopolymer materials [3,10].

Generally, it can be stated that the application of EP to the concrete mix instead of natural aggregates leads to the reduction of the hardened concrete strength as the perlite content in the concrete mix increases [11,12,13,14]. The above is explained by the introduction of the porous aggregate with a low strength to the concrete mix. Reduction of the concrete strength was also found in case of ground EP applied as the additive to cement in the concrete mix [15,16,17]. It was explained by a significant impact of ground expanded perlite on the loss of concrete mix fluidity. Therefore, despite using superplasticizers, at low water to binder ratios (<0.35) the hardened concrete showed increased porosity and therefore lower density and strength [18]. As per test results, application of the ground EP as an additive to cement may also increase the strength of the hardened concretes [19,20]. This may be explained by the fact that EP ground to the particle size characteristic of cement is able to to activate the pozzolanic properties of expanded perlite. Consequently, the content of C-S-H phase formed in the reaction of portlandite with EP increases, and thus, the porosity of the interfacial transition zone (ITZ) between aggregate and cement matrix in hardened concretes is reduced [21].

Many authors conducted research on lightweight ultra hight performance concerts (LUHPC) [22,23,24]. It was demonstrated that it is possible to achieve LUHPC with compressive strength ranging from 35 to 70 MPa, with a density ranging from 1440 to 1840 kg/m^3^, with the addition of pozzolan materials such as silica fume, fly ash, metakaolin, volcanic ashes, calcined clays, and shales [22]. During the determination of the usefulness of the lightweight concrete as a structural material, it is very important to define its flexural and tensile strength. While testing the LUHPC, the authors of the paper [22] proved that lightweight concrete with lower density demonstrated higher flexural strength than concrete with the same compressive strength but higher density. The authors of the paper [25] demonstrated the possibility of increasing flexural strength by incorporating steel microfibers into concrete mix, achieving concrete with a density of approximately 1700 kg/m^3^ [25]. Moreover, they stated that the introduction of fibers in amounts up to 0.75 vol.% does not cause significant workability loss in the concrete mix. With the aim of achieving lightweight concrete with lower density, containing lightweight aggregates while also demonstrating good mechanical properties, the authors of papers [26,27] introduced basalt fibers in lightweight concretes. However, the addition of those fibers to the concrete mix decreased its workability significantly.

It is necessary to point out that numerical methods, applied to estimate the behavior of structure elements under loads, are very helpful to analyze the tensile strength of concrete or to predict areas and intensity of crack formations [28]. However, the application of these numerical methods to modeling the lightweight concrete, composed as they are with materials of very different elastic modulus, is more complicated compared to normal concrete or high performance concrete [29].

The literature contains very few studies dedicated to the implementation of reactive powder concrete (RPC) in lightweight concretes technology. Sadrekarimi [30] obtained the lightweight RPC with density ca. 1900 kg/m^3^ and the height compressive strength (ca. 280 MPa), by simply increasing the content of silica fumes in the RPC composition, without the use of lightweight aggregate, while applying the increased temperature (240 °C) during 24 h of concrete curing. The author also showed that the concretes cured in temperatures of 240 °C are characterized by lower density compared to the concretes cured in temperatures of 90 °C [30].

Gökçe et al. [31] demonstrated that it was possible to obtain lightweight RPC with density of 1840 kg/m^3^ and the compressive strength of ca. 69 MPa when quartz was replaced with a lightweight volcanic aggregate (2400 kg/m^3^). Achieving higher compressive strength was possible with the application of lightweight aggregates, with the parallel application of steel micro fibers and pressure curing. However, such a treatment increased the RPC density to about 2400 kg/m^3^ with an increase of the compressive strength up to 176 MPa and a decrease of water absorption from 6.5% (for concrete cured in ambient conditions) down to 1.7% at the same time. 

Studies [32,33] applied lightweight aggregates such as Pollytag and expanded clay [32] or expanded polystyrene beads-EPS [32,33]. The results showed that it was possible to obtain lightweight RPC without the application of thermal or pressure treatment, with the compressive strength in the range of ca. 17 MPa to ca. 83 MPa after 28 days, depending on the type and quantity of lightweight aggregate used. It was found that the addition of expanded clay to RPC had no impact on the increase of water absorption of this lightweight RPC, compared to the reference RPC without lightweight aggregate [32]. However, water absorption of RPC with Pollytag and expanded polystyrene beads-EPS is higher by about 1.5% compared to RPC without lightweight aggregates [32].

The research into lightweight reactive powder concrete with perlite was conducted by Al-Jumaily [34]. The author showed that with the increase in perlite content in the RPC mix, in quantities from 2.5% to 10% by cement mass, the compressive strength and elastic modulus decrease. After 28 days, the compressive strength, with 2.5% of perlite in the RPC, equals 82.5 MPa, and for higher content of perlite (10%) the compressive strength equals 53.9 MPa, whereas the elastic modulus is 43.1 GPa and 31.0 GPa, respectively. Contrary to the findings of the authors of this paper, the author of [34] demonstrated that increasing the content of perlite in the RPC mix up to 10% causes the water absorption rate to increase by approximately 2%.

In this study, the influence of expanded perlite in quantities of 30%, 45%, and 60% by volume to the RPC mix, on the lightweight RPC properties was tested. Particular attention was paid to changes in density, compressive strength, and water absorption with the increase in quantity of EP in RPC. Microstructure analyses were also conducted, as well as investigation into the influence of pozzolanic activity of EP on the compressive strength of lightweight RPC.

## 2. Materials and Methods

For testing, the RPC mix was prepared with the blast furnace slag cement-CEM III/A 42.5 N, (specific surface area of cement acc. to Blaine was 360 m^2^/kg), with GGBFS content in amounts of ca. 60% by mass. Finely ground quartz sand, with maximum grain size < 800 μm, quartz powder, and waste silica, with the content of SiO_2_ > 90% by mass, were used. Theater to binder ratio equaled 0.2 and was obtained by the use of the polycarboxylate superplasticizer in the amount of 3.0% of the cement mass. The research of the chemical and physical properties (such as chemical composition and particle size distribution) of the RPC mix composition are presented in this paper [32]. The optimalization of the RPC mix components, in order to increase the particles packing, was carried out based on a Funk and Dinger curve [35], as well as on the experience of Zdeb and Śliwiński [36].

**Figure 1:**
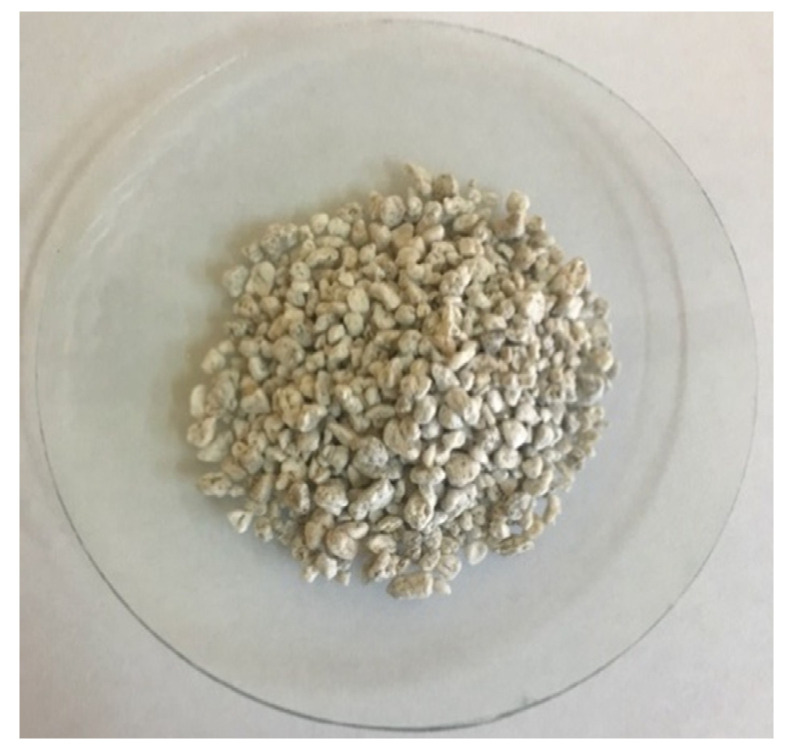
Image of expanded perlite.

RPC mixes with perlite added in the amounts of 30%, 45%, and 60% by volume of the concrete mix were prepared to be tested. The expanded perlite of 0/4 mm fraction was used (Figure 1). The particle size distribution of the lightweight aggregate used is given in Table 1.

Expanded perlite (0/4 fraction) primarily contains grain sizes >2 mm (52%). The aggregate used contained ca. 4% of powder with the particle size <0.063 mm. 

Increasing the quantity of expanded perlite (EP) in the RPC mix caused significant deterioration of its fluidity and required an increase in the amount of a superplasticizer from 3.0% to 5.0% by mass, at 60% of the EP content. The composition of tested mixes is presented in Table 2. The procedure of mixing the ingredients was maintained for all mixes as follows: to begin with, the dry ingredients were mixed (cement, waste silica, quartz powder, and quartz sand). Then water was added, followed by the superplasticizer. When the homogeneous and fluid RPC mix was obtained, the lightweight aggregate was added.

The tests of the physical properties of EP (the loose bulk density, the water absorption, and the resistance to crushing), were performed in accordance with PN-EN 13055:2016-07 standard—“Lightweight aggregates”.

The consistence of the lightweight RPC mixes was tested by means of the flow table method, in accordance with PN-EN 1015-3:2000—“Methods of test for mortar for masonry—Part 3: Determination of consistence of fresh mor-tar (by flow table)”, by measuring the concrete flow diameter after 10, 30, and 60 min. 

The tests of concrete density were conducted on the samples sized 150 × 150 × 150 mm^3^ according to PN-EN 12390-7:2011—“Testing hardened concrete—Part 7: The density of hardened concrete”.

The tests of water absorption were carried out for 28 days after the samples’ preparation. The sample size was 100 × 100 × 100 mm^3^. The samples were saturated with water and then dried to constant mass at the temperature of 50 °C. The test procedure is described in Polish standard PN-B 06250:1988 “Ordinary concrete”.

To test strength, the prism samples were prepared sized 40 × 40 × 160 mm^3^. After mix preparation, and casting them in steel molds, the samples were demolded after 24 h and cured in water at a temperature of 20 ± 2 °C until the strength test (i.e., for 365 days). The procedure of flexural and compressive strength testing is described in PN-EN 196-1:2016-07—“Methods of testing cement—Part 1: The determination of strength”.

The freeze-thaw resistance of lightweight RPC in the presence of de-icing salt was tested by means of Slab test (PKN-CEN/TS 12390-9:2017-07—“Testing hardened concrete—Part 9: Freeze-thaw resistance with de-icing salts. Scaling”), the classification of the concrete under its freeze-thaw resistance was based on a Swedish standard SS 13 72 44 “Concrete testing; Hardened concrete; Scaling at freezing—2005”.

The microstructure of lightweight RPC was carried out with the use of the NOVA NANO SEM 200 scanning microscope (FEI Europe B.V., Eindhoven, The Netherlands) with the possibility of obtainment the EDS spectra in selected points.

The test of the phase composition of EP and hardened lightweight RPC were conducted by means of powder X-ray diffraction within the angle range of 5° to 60° 2θ. The Philips X’PertSystem diffractometer (Amsterdam, The Netherlands) and CuKα radiation was used. The JCPDS-ICDD database was used to define the crystalline minerals based on known patterns [37].

## 3. Results

### 3.1. Physical Properties and Phase Composition of Expanded Perlite

**Figure 2:**
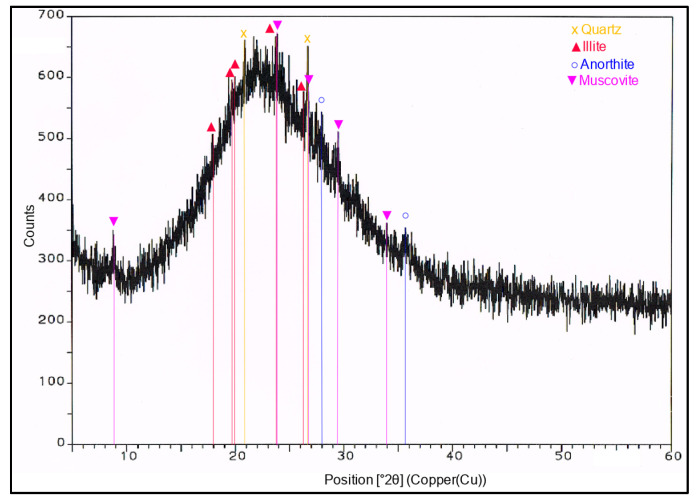
XRD pattern of expanded perlite.

The results of the physical properties tests for expanded perlite are presented in Table 3, whereas the diffractogram of the expanded perlite specimen is shown in Figure 2.

**Figure 3:**
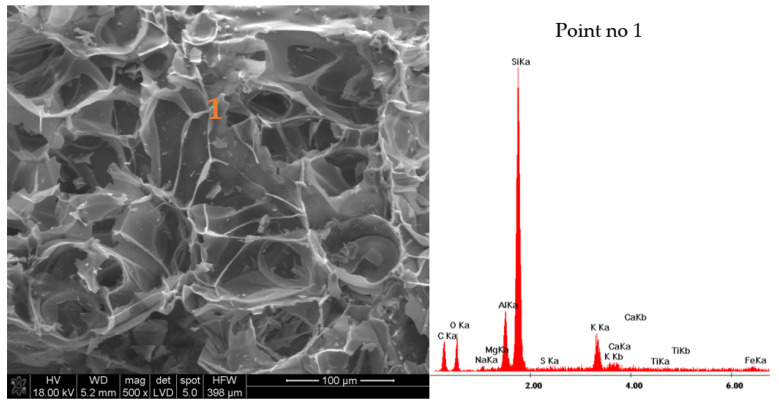
Microstructure of expanded perlite grain in 500 times magnification with EDS analysis in point 1.

Based on the results of the tests of the EP phase composition conducted by means of X-ray powder diffraction (Figure 2), it can be seen that the expanded perlite consists mainly of the glassy phase, which is proven by the increase in the background on the specimen diffractogram. The crystal phases in the form of quartz and aluminosilicates; illite, muscovite, and anorthite containing potassium in their structure occur in small amounts. The grains of the expanded perlite have a very developed surface with numerous open pores (Figure 3). Based on the EDS spectrum obtained for the field in point 1 in Figure 3, it can be stated that the perlite contains mainly silicon, then aluminum, and potassium. There are also small amounts of magnesium, sodium, and iron in the perlite phases.

### 3.2. Consistence of Concrete Mixes

**Figure 4:**
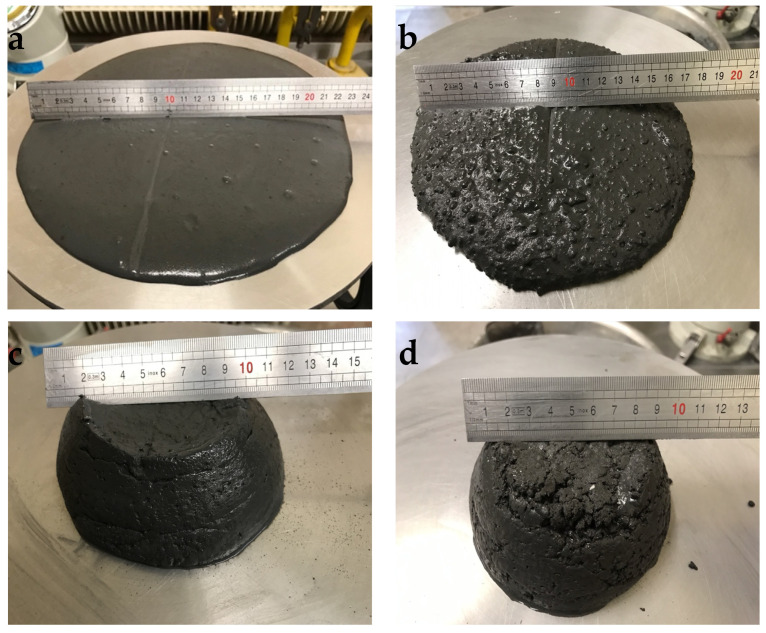
Flow diameter of RPC mix without lightweight aggregate (**a**), and containing 30% EP (**b**), 45% EP (**c**), and 60% EP (**d**).

Table 4 and Figure 4 show consistence test results of RPC mixes containing 30%, 45%, and 60% by volume of expanded perlite and RPC mix without lightweight aggregate.

It has been found that the addition of expanded perlite to RPC causes the loss of mix fluidity. Introduction of 30 vol.% of lightweight aggregate causes a reduction in the mix flow diameter from 220 mm to 175 mm. Application of larger quantities of EP (45% and 60%), despite the increased amount of superplasticizer in the concrete mix to 4.0% and 5.0% by mass, respectively, caused further significant loss of fluidity. The flow diameter of RPC mix with the application of 60 vol.% of expanded perlite was only 95 mm after 1 h, and at EP content of 45 vol.% it was 125 mm. 

The deterioration of concrete workability due to the introduction of expanded perlite was shown by the authors of papers [38,39]. This phenomenon was explained by water absorption from the mix by porous aggregate. This causes a decrease in the effective water/cement ratio [40], and consequently leads to a reduction in concrete durability [41].

There are also reports showing that the workability of concrete improves by adding EP [11,13,42]. Authors of papers [3,43] claim that the improvement of workability is associated with the increase in air content in the concrete mix, due to the addition of the very porous aggregate, which is expanded perlite. It was also shown that the application of the lightweight aggregate saturated with water caused improvement in mix workability, along with an increase in expanded perlite content in the mix [3,43,44].

### 3.3. Properties of RPC with Expanded Perlite

#### 3.3.1. Distribution of Expanded Perlite in RPC

**Figure 5:**
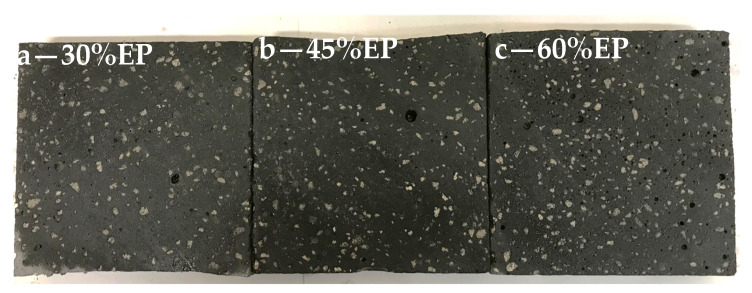
Distribution of EP in the structure of lightweight RPC 30% EP (**a**), 45% EP (**b**), and 60% EP (**c**).

Expanded perlite is distributed evenly in the concrete mix, which is visible in macroscopic images of lightweight concrete samples containing 30%, 45%, and 60% of expanded perlite by volume after 28 days of curing (Figure 5). The high plastic viscosity of the RPC mix, which prevents the segregation of ingredients, has an impact on the even distribution of the aggregate in the concrete mix [32].

#### 3.3.2. Density

**Figure 6:**
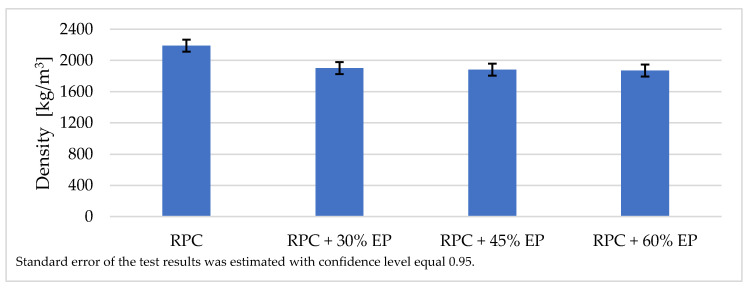
Density of RPC and density of RPC containing EP in quantities 30%, 45%, and 60% by volume.

As expected, the addition of the expanded perlite to the RPC mix in quantities from 30 vol.% to 60 vol.% causes a reduction in the concrete density by approximately 13%, compared to the density of the RPC without lightweight aggregate (Figure 6). It was found, however, that application of larger quantities of EP to the concrete mix (45% and 60% by volume) caused a slight reduction in concrete density (Figure 6). Concrete density with 30 vol.% content of lightweight aggregate is 1900 kg/m^3^, whereas with the content of 45 vol.% and 60 vol.%, it is 1880 kg/m^3^ and 1870 kg/m^3^, respectively, and it differs slightly from the density of concrete containing 30 vol.% of EP.

The reason for this phenomenon is the low crushing resistance of expanded perlite. Therefore, the lightweight aggregate, especially in higher dosage, requires a longer mixing time when being mixed with the RPC mix, in order to obtain the homogeneous and fluid concrete mix. Therefore, this proves that the application of this aggregate in larger quantities (45% and 60%) is not recommended to obtain the lightweight RPC with expanded perlite, as it does not allow us to obtain the concrete with a lower density.

The reduction in concrete density may be achieved by the application of expanded perlite as a substitute to natural aggregate or sand, which has a specific gravity much lower than the specific weight of other ingredients of the RPC mix [11,38,45]. The authors of the paper [34] showed that the application of perlite to RPC in quantities from 2.5% to 10% by cement mass allowed the reduction in the weight of that concrete from 4.5% to 19.3%.

#### 3.3.3. The Compressive and Flexural Strength

**Figure 7:**
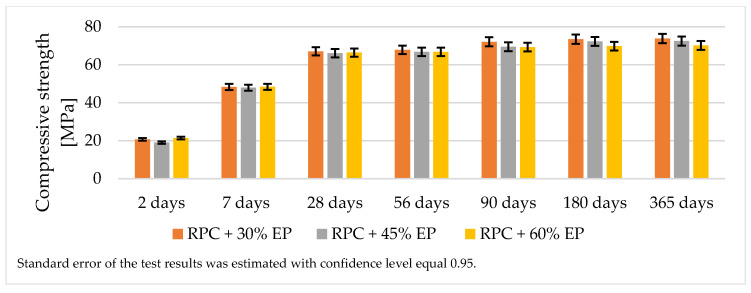
Compressive strength of lightweight RPC containing EP in quantity 30%, 45%, and 60% by volume.

**Figure 8:**
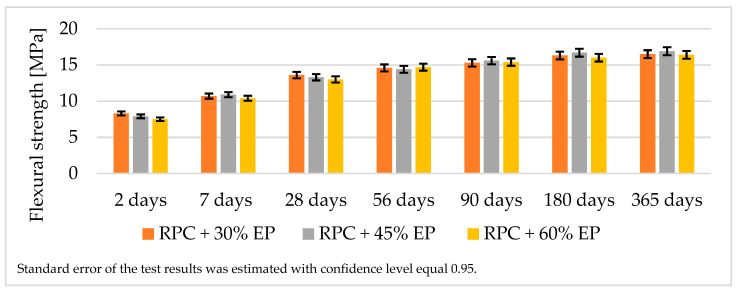
Flexural strength of lightweight RPC containing EP in quantity from 30%, 45%, and 60% by volume.

Figure 7 and Figure 8 show test results of the compressive and flexural strength of lightweight concretes containing expanded perlite.

The RPC based on blast furnace slag cement CEM III/A 42.5 N used in this study achieves the compressive strength equal to 128.3 MPa after 28 days, and equal to 175.2 MPa after 365 days, and the flexural strength equal to 22.6 MPa after 28 days and 27.5 MPa after 365 days [46]. A high increase in the compressive strength of the blast furnace slag cement-based RPC (by ca. 47 MPa) takes place between the 28th and the 365th day of curing, and it is associated with a significant quantity of the ground granulated blast furnace slag in the cement (approximately 60% by mass).

The analysis of the strength test results for lightweight RPC containing 30 vol.% of expanded perlite showed that it was possible to obtain the concrete with a compressive strength > 65 MPa after 28 days and >70 MPa after 365 days of curing (Figure 7). The flexural strength of the lightweight RPC containing EP in quantity 30% by volume is approximately 16.5 MPa after 365 days (Figure 8.). The increase in the compressive strength of the concrete between the 28th and the 365th day of curing is not significant (just a few MPa). Based on the results of the compressive strength of concretes containing increased quantities of EP (45 vol.% and 60 vol.%), it can be stated that the values of the compressive strength of these concretes are similar to the compressive strength of the concrete containing 30 vol.% of EP.

The compressive and the flexural strength values for RPC with expanded perlite after 28 days is approximately 40% of the compressive strength value of the same concrete without the addition of the lightweight aggregate. We maintained the RPC strength at more or less the same level with various content of aggregate (30%, 45%, and 60%). The similar values between the compressive and the flexural strength of RPC containing 30 vol.%, 45 vol.%, and 60 vol.% of EP is due to the fact that the aggregate becomes finer during the longer mixing time which the components with the increased amount of perlite require to obtain the homogeneous and fluid concrete mix. This explains why the increased amounts of EP (45 vol.% and 60 vol.%) in RPC do not cause a reduction in the lightweight RPC density, which stays at the same level as RPC with 30 vol.% of EP. Expanded perlite shows a very low crushing resistance (0.66 MPa), compared to an aggregate with expanded clay (2.24 MPa) or an aggregate made of processed fly ash (3.83 MPa) [32]. However, the authors of the cited papers observed that, with increased addition of EP to cement based elements [43], high strength concrete [11], and self-compacting concretes [40] the compressive strength value decrease is parallel with the decrease in density.

#### 3.3.4. The Water Absorption and Freeze-Thaw Resistance

The test results of the water absorption and the scaling mass of concrete samples subjected to freezing and de-icing salt are presented in the Table 5.

The water absorption of RPC equals only 3.3%. The received results show that water absorption of RPC with EP (Table 5) slightly differ from the water absorption of pure RPC. A relationship between an increased amount of EP in RPCC mix and water absorption was not observed. 

The results of freeze-thaw resistance of lightweight RPC in the presence of de-icing salt (Table 5), showed that, according to the classification presented in Swedish standard SS 13 72 44, tested concretes may be classified as a concrete characterized by very good resistance to the impact of the de-icing salt. The scaling mass of the lightweight RPC samples is slightly higher in comparison to RPC without EP. The above may be explained by the exposure of the concrete surface with uncovered aggregate, which demonstrates a lower freeze-thaw resistance then very dense RPC matrix, to freeze-thaw cycles in the presence of de-icing salt.

#### 3.3.5. Microstructure and Phase Composition

**Figure 9:**
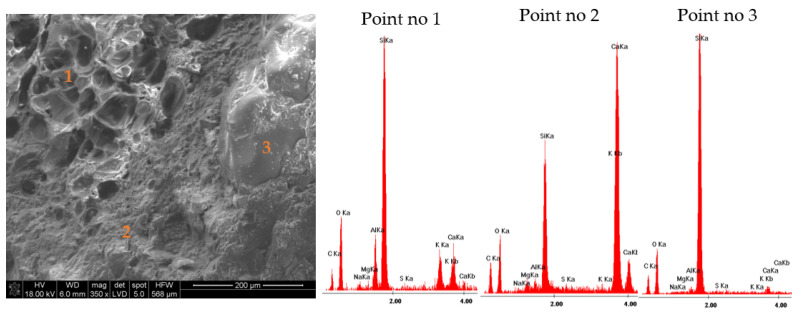
Microstructure of lightweight RPC with 30 vol.% of EP, cured for 28 days in 350 times magnification with EDS analysis in point 1, 2, and 3.

**Figure 10:**
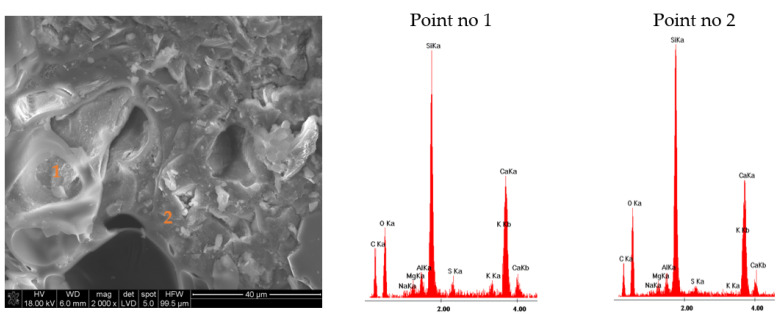
Microstructure of lightweight RPC with 30 vol.% of EP, cured for 28 days in 2000 times magnification with EDS analysis in point 1 and 2.

**Figure 11:**
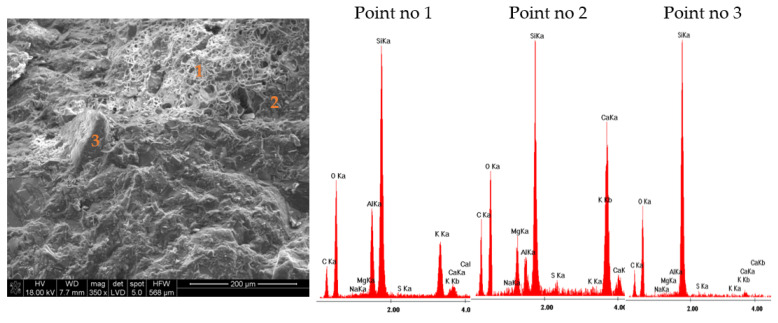
Microstructure of lightweight RPC with 60 vol.% of EP, cured for 28 days in 350 times magnification with EDS analysis in point 1, 2, and 3.

**Figure 12:**
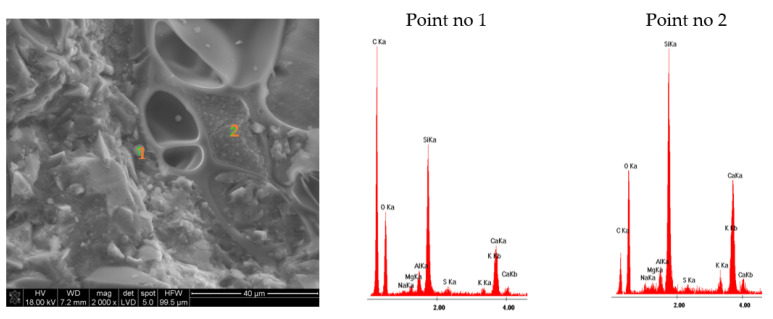
Microstructure of lightweight RPC with 60 vol.% of EP, cured for 28 days in 2000 times magnification with EDS analysis in point 1, 2, and 3.

The results of the microstructure tests of the lightweight RPC with expanded perlite in quantities of 30% and 60% by volume are presented in Figure 9 and Figure 10 (RPC + 30% EP) and Figure 11 and Figure 12 (RPC + 60% EP). The EDS spectra were performed for selected points. No significant differences are observed in the microstructure of the concrete with 30 vol.% of EP as compared to the concrete containing 60 vol.% of the aggregate. In Figure 9 and Figure 11, the compacted C-S-H phase is visible (EDS in point 2, Figure 9 and EDS in point 2, Figure 11) closely adhered to grains of perlite and quartz. No clear ITZ is visible. The concrete microstructure is compact, without larger pores in the RPC matrix visible. At larger magnification (Figure 10 and Figure 12), the C-S-H phase is visible in perlite pores. This is confirmed by EDS spectra made in points 1 and 2, Figure 10, and in points 1 and 2, Figure 12. In Figure 12 of the RPC containing 60% of EP, an area with partially ground EP can be seen (a lack of large surface pores of aggregate), which is not observed in the microstructure of the RPC containing 30 vol.% of EP. The analysis of the EDS spectra in points 1 and 2 in Figure 12 confirms the presence of elements in this area, which belong to the aluminosilicate phases occurring in the expanded perlite.

The concrete mix location in aggregate pores strengthens the bond between the aggregate and the RPC matrix. The bonding between the lightweight aggregate and the cement paste may be created by mechanical bonding, absorption of water and cement paste from the fresh concrete mix by the aggregate or, in some cases, the pozzolanic reactivity of the lightweight aggregates [47,48]. Due to the confirmed pozzolanic properties of expanded perlite, a reaction of that aggregate with calcium ions originating from cement hydration is possible, as is the strengthening of the bond between the RPC matrix with the lightweight aggregate [49]. Based on the EDS analysis (Figure 11, point 2 and Figure 12, points 1 and 2) it can be stated that the C-S-H phase has a low CaO/SiO_2_ ratio. It is undoubtedly the result of the blast furnace slag cement application containing approximately 60% of the ground granulated blast furnace slag (GGBFS). The CaO/SiO_2_ ratio in the C-S-H phase for pastes with GGBFS addition may amount to 1.2, whereas for pastes of Portland cement, this ratio is higher and may even amount to 1.9 [50].

**Figure 13:**
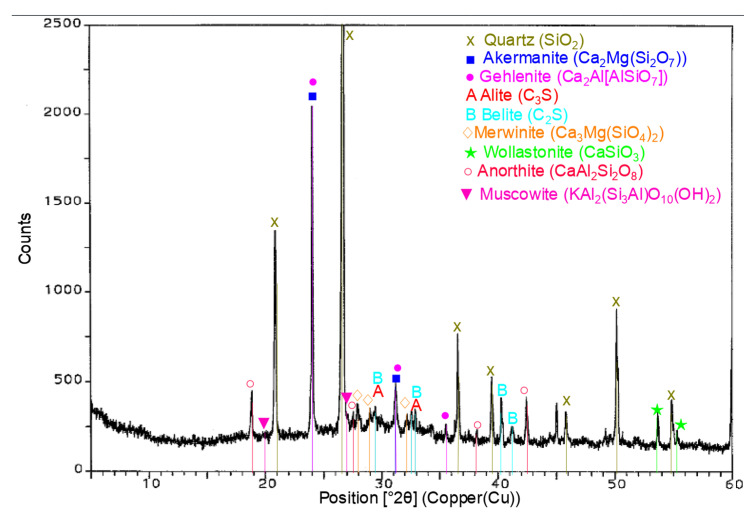
XRD RPC +30% EP after 28 days of curing.

**Figure 14:**
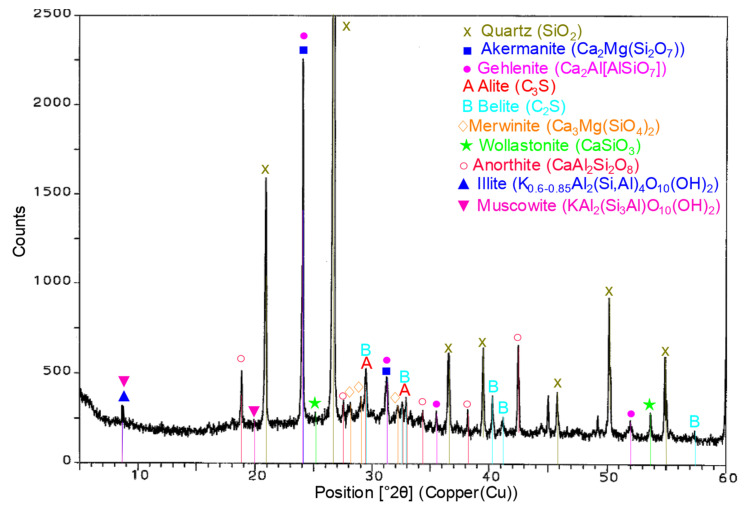
XRD RPC + 60% EP after 28 days of curing.

The analysis of the XRD patterns for the RPC specimens containing the expanded perlite in quantity of 30% (Figure 13) and 60% by volume (Figure 14), after 28 days of hydration, apart from peaks belonging to clinker phases (alite and belite), showed the presence of low intensive peaks from the lightweight aggregate such as illite and muscovite (Figure 13 and Figure 14). Increasing the quantity of expanded perlite in RPC (60 vol.%) causes the appearance of the intensity of peaks characteristic for muscovite (d = 9.9705 Å and d = 3.1997 Å).

It is known that the finely ground expanded perlite added to cement shows pozzolanic properties when reacting with calcium ions and alkalis [4]. As early as in the first few minutes of the cement hydration process, the ions Ca^2+^, K^+^, N^+^, and OH^−^ are adsorbed on the surface of the reactive aggregates [51]. In case of a perlite pozzolanic reaction, it is beneficial to introduce this material with at least the same level of fineness as of the cement [4].

The tests of the expanded perlite particle size distribution conducted in this study (Table 1) showed that the content of fraction < 250 μm in this aggregate was ca. 17%. It was demonstrated that, during longer periods of mixing time of the RPC components with the EP, the lightweight aggregate becomes finer, which in consequence results in the increased content of fine particles.

The tests of the expanded perlite pozzolanic activity conducted in this study demonstrated that the expanded perlite showed significantly lower pozzolanic activity compared to fly ash, silica fume, and even quartz powder (Table 6). This is undoubtedly associated with the large size of the expanded perlite grains [4]. Tests of pozzolanic activity of the EP were conducted with the application of a procedure for testing fly ash pozzolanic activity according to PN-EN 450 standard for expanded perlite added to RPC mixes. For tests of pozzolanic activity, the perlite was used in the same volume as fly ash used for the cement replacement for similar tests.

#### 3.3.6. Impact of Calcium Hydroxide Addition on Concrete Strength

**Figure 15:**
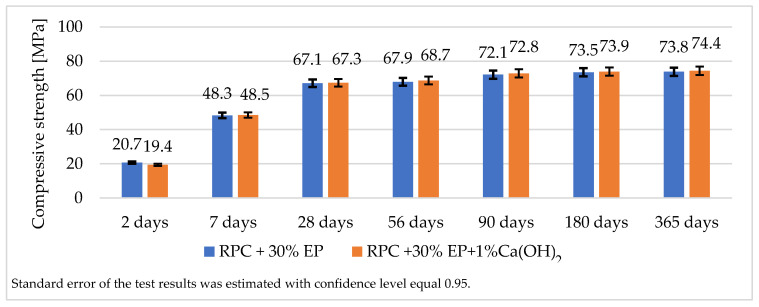
Compressive strength of RPC containing 30 vol.% of expanded perlite without and with addition of 1% Ca(OH)_2_.

Taking the pozzolanic properties of the expanded perlite into account, tests were undertaken of with lightweight RPC containing 30% of perlite, with the addition of hydrated lime in 1% of the cement mass to the RPC mix. Data presented in Figure 15 show that the compressive strength obtained for specimens containing lime is higher by only a few tenths of MPa than the strength of the samples without this additive.

**Figure 16:**
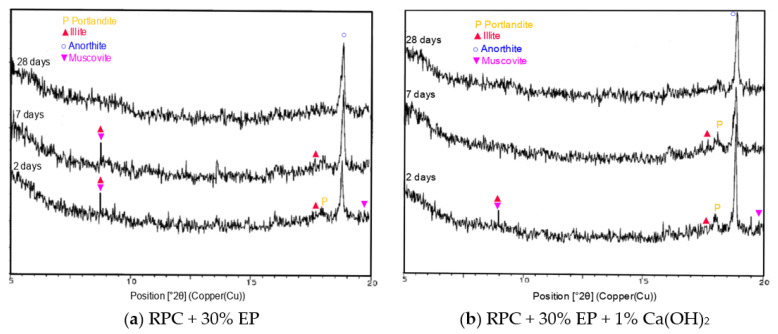
XRD patterns: (**a**) RPC +30% EP after 2, 7, and 28 days of curing, (**b**) RPC + 30% EP + 1% Ca(OH)_2_ after 2, 7, and 28 days of curing.

The analysis of the XRD patterns for the concrete specimens containing the expanded perlite in the amount of 30 vol.%, with the addition of 1% of calcium hydroxide (Figure 16), showed that, along with the progress of hydration (after 28 days), a slight peak characteristic for portlandite (d = 4.9093 Å), visible after 2 and 7 days of hydration, disappears. Peaks characteristic for portlandite do not occur on the diffractograms of the specimens of the lightweight RPC without lime addition from the 7th day of hydration (Figure 16). Later (after 28 days), on the diffractogram of the RPC + 30% EP + 1% Ca(OH)_2_ sample, the peaks characteristic for muscovite, one of the expanded perlite phases, disappear.

## 4. Discussion

It is well known that expanded perlite may be used, in powder form, as a mineral additive to cement, where the advantages of its pozzolanic properties may be utilized [21]. However, EP which is not ground is used as a lightweight aggregate, and it was in this form utilized to RPC in this paper.

The results of consistency tests of RPC mix with EP showed that increasing the content of aggregate in an RPC mix causes significant workability loss (Table 4). After 10 min, the flow diameter of RPC containing 60 vol.% of EP is almost two times shorter than the flow diameter of RPC without lightweight aggregate. The slight decrease in flow diameter over a period of 1 h suggests that the workability of the mix remains at approximately the same level. Similar phenomena are observed in RPC mixes with a lower content of EP (30 and 45 vol.%). The amount of EP in an RPC mix not only has an impact on the significant workability loss, but also the fact that expanded perlite is characterized by a low resistance to crushing (0.66 MPa) and, during mixing, all ingredients of an RPC mix are ground. The elements of ground EP were observed on microscopic images (SEM) of hardened lightweight RPC (Figure 11). 

**Figure 17:**
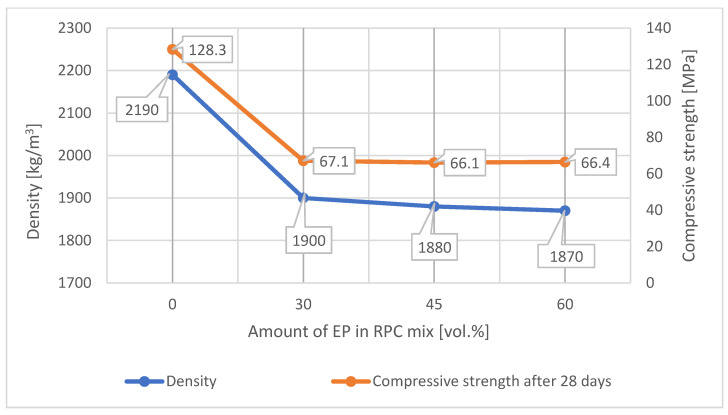
Change in density and compressive strength depending on the amount of EP in the RPC.

A longer period of mixing time of an RPC mix which shows high viscosity [32], which is required to achieve a homogeneous and fluid concrete mix, causes the grinding down of the expanded perlite. Therefore, the increase of EP in RPC above 30 vol.%, does not cause a significant decrease in density, and compressive strength remains at approximately the same level (Figure 7, Figure 8 and Figure 17). Such an occurrence is not observed in RPC with lightweight aggregates, which represent higher resistance to crushing (Pollytag and expanded clay aggregate), as well as with expanded polystyrene beads (EPS) [32]. In the case of the abovementioned lightweight aggerates, the increase in aggregates in RPC from 30 vol % to 60 vol.% leads to a decrease in the density of hardened lightweight RPC from ca. 6% to 15%. This observed phenomenon of the grounding down of expanded perlite in RPC mix allows us to state that utilization of higher amounts of EP (45 and 60 vol.%) in RPC is not recommended, bearing in mind the obtainment of lightweight concrete.

It is notable that conducted analysis by the authors of this paper of the research presented in a previous paper [34], concerning the density of lightweight RPC including perlite with the increase in its participation in RPC mix, allows us to state that, also in this case, a significant decrease in lightweight RPC density with increase in perlite in the mix, in range from 2.5 to 10 mas.%, is not observed. However, the author of the paper [34] does not comment on this phenomenon. 

The fact that ground EP shows pozzolanic properties [21] prompted the authors of this paper to conduct the research concerning the pozzolanic properties of this material in RPC mix. The compressive strength tests, which were canaried out on RPC containing 30 vol.% of EP with addition of 1.0% by cement mass of hydrated lime, did not show an evident strength increase in the material (the compressive strength increased just a few tenths of an MPa). The above emphasizes the lack of significant impact the ground EP pozzolanic properties have on the lightweight RPC compressive strength. However, phase composition tests of hardened lightweight RPC containing 30 vol.% of EP, with the addition of 1.0% hydrated lime and cured for 28 days (Figure 17), showed the disappearance of characteristic reflections for portlandite and muscovite, the identified phase in EP (Figure 2), proving that the pozzolanic reaction between EP and calcium ions occurs.

Yao et al. [52] demonstrated that muscovite is a mineral able to react with calcium hydroxide, forming the C-S-H gel with potassium cations built in. These authors also showed that grinding the perlite had a significant impact on the pozzolanic activity of component minerals, including the pozzolanic activity of muscovite. Taking the above statements and the results of our own study into consideration, a slight increase in the compressive strength of the lightweight RPC with 30% EP and the addition of 1% hydrated lime, as compared to the compressive strength of the concrete without this additive, may be associated with the pozzolanic reaction of the silicate ions originating from the perlite phases with the calcium ions (Ca^2+^) additionally introduced to the RPC mix.

The pozzolanic properties of the expanded perlite are revealed to a greater extent when the ground perlite is added to the cement [21]. Its addition clearly reduces the quantity of portlandite in hydrated cement pastes. However, the pozzolanic activity of expanded perlite is slightly lower than the activity of fly ash (Table 6). Authors of the paper [53] used expanded perlite with other pozzolanic additives, such as fly ash, and demonstrated that in these configurations the perlite showed almost inert pozzolanic properties. The compressive strength of the mortars where 10% of fly ash was replaced with perlite, after 28 days, reduced by half, as compared to mortars with fly ash.

RPC with EP in quantity from 30% to 60% by volume are characterized by very low water absorption, ranging from 3.3% to 3.5%, which is not significantly different than the water absorption of RPC without a lightweight aggregate (3.3%). The above was explained by the very compact RPC microstructure, without visible larger pores (Figure 9 and Figure 11). The C-S-H phase precisely adheres to quartz and EP grains, also penetrating lightweight aggregate’s pores (Figure 10 and Figure 12). The consequence of such a compacted lightweight RPC microstructure is presented in the paper as an excellent resistance to freezing and thawing cycles in the presence of de-icing salt. RPC with EP, according to classification presented in Swedish standard SS 13 72 44, may be considered as concrete with very good freeze-thaw resistance. 

## 5. Conclusions

Introduction of the expanded perlite-fraction 0/4 mm-to the RPC mix in quantities from 30% to 60% by volume causes a reduction in mix fluidity and homogeneity, which increases as a larger content of aggregate is applied in the mix. Maintaining the mix fluidity while using the larger amount of EP requires a larger quantity of a superplasticizer (to 5% by mass) and a longer time to mix ingredients.

EP’s low resistance to crushing and required longer mixing time of the mix components causes grounding down the expanded perlite, which therefore has no influence on the decrease in the lightweight RPC density.

The addition of EP to RPC mix in the quantity of 30 vol.%, causes a decrease in the concrete density from 2200 kg/m^3^ to 1900 kg/m^3^. However, a further increase in EP content in RPC mix, from 30 to 60 vol.%, results in a lack of decrease in density and compressive strength of those lightweight concretes, which should have been expected with a significant increase in EP in the RPC. This is the result of the weak aggregate getting finer due to its very low resistance to crushing. The above test results indicate that application of larger amounts of EP (45 and 60 vol.%) is not recommended in order to obtain lightweight RPC. 

The water absorption of lightweight RPC with EP (30, 45, and 60 vol.%) is in the range of 3.3% to 3.5%, and is comparable to the water absorption of RPC without lightweight aggregate (3.3%). The low water absorption of lightweight concrete was explained by very compacted microstructures of hardened lightweight RPC.

Expanded perlite in the form of aggregate shows pozzolanic properties. Its pozzolanic activity, however, is much lower than the activity of silica fume and quartz powder, the ingredients of the RPC mix. It has been demonstrated that EP in the RPC mix reacts with calcium hydroxide, but the pozzolanic activity it shows has a marginal impact on the increase of the lightweight RPC strength.

## Figures and Tables

**Table 1:** Gradation of expanded perlite.

Percentage Pass [% Mas.]							
Sieve size [mm]	0	0.063	0.25	0.5	1.0	2.0	4.0
Expanded perlite (EP)	0	4	17	27	32	48	100

**Table 2:** Composition of RPC mix with expanded perlite (kg/m^3^).

Component	Cement	Expanded Perlite (EP)	Waste Silica	Quartz Powder	Quartz Sand	w/c	w/b	SP ^1^
RPC without lightweight aggregate	850.0	-	170.0	289.0	688	0.24	0.20	3%
RPC + 30% EP	724.0	52.8	144.8	246.0	586.4			3%
RPC +45% EP	707.0	78.4	141.4	240.4	572.7			4%
RPC +60% EP	693.0	102.6	138.6	235.6	561.3			5%

^1^ SP—superplasticizer.

**Table 3:** Physical properties of expanded perlite.

Sample	Loose Bulk Density [kg/m^3^]	Water Absorption [%]	Crushing Resistance [MPa]
Expanded perlite (EP)	123.3	19.3	0.66

**Table 4:** Flow diameter of lightweight RPC mixes for 60 min (mm).

Sample	10 min	30 min	60 min
RPC without lightweight aggregate	220	225	225
RPC + 30% EP	175	170	175
RPC + 45% EP	130	125	125
RPC + 60% EP	115	110	95

**Table 5:** Water absorption and scaling during freeze-thaw test of lightweight RPC.

Sample	RPC	RPC + 30% EP	RPC + 45% EP	RPC + 60% EP
Water absorption [%]	3.3	3.3	3.5	3.4
Scaling [kg/m^2^]	0.026	0.040	0.044	0.040

**Table 6:** Pozzolanic activity of mineral additives.

Sample	Pozzolanic Activity after 28 Days [%]	Pozzolanic Activity after 90 Days [%]
Fly ash	76.7	87.2
Silica fume	91.5	95.8
Quartz powder	71.6	75.9
Expanded perlite (EP)	49.2	63.2

## Data Availability

The data presented in this study are available only in “Lightweight Reactive Powder Concrete Containing Expanded Perlite”.
